# ^18^F-FDG PET/CT metabolic parameters are correlated with clinical features and valuable in clinical stratification management in patients of castleman disease

**DOI:** 10.1186/s40644-025-00833-9

**Published:** 2025-02-12

**Authors:** Guolin Wang, Qianhe Xu, Yinuo Liu, Huatao Wang, Fei Yang, Zhenfeng Liu, Xinhui Su

**Affiliations:** Department of Nuclear Medicine, The First Affiliated Hospital, Zhejiang University School of Medicine, Hangzhou, 310003 China

**Keywords:** Castleman disease, ^18^F-FDG PET/CT, metabolic parameters, Spleen to liver ratio (SLR), Disease severity

## Abstract

**Background:**

Castleman disease (CD) is a rare lymphoproliferative disorder. This study is to evaluate the correlation between ^18^F-flurodeoxyglucose (^18^F-FDG) positron emission tomography-computed tomography (PET/CT) and clinical features in CD patients, and exploring its value in distinguishing disease severity and assisting in risk stratification.

**Methods:**

We retrospectively enrolled 93 patients with newly diagnosed CD. Traditional semi-quantitative ^18^F-FDG PET/CT parameters including the maximum standardized uptake value (SUV_max_), total metabolic lesion volume (MLV), total lesion glycolysis (TLG) were measured, and the lymph node to liver ratio of SUV_max_ (LLR), lymph node to mediastinal blood pool of SUV_max_ (LMR), spleen to liver ratio of SUV_max_ (SLR) and No. of involved lymph node stations (LNS) were calculated. The correlation between these metabolic parameters and clinical features were studied using a univariate analysis. The influencing factors of CD severity were determined by univariate and multivariate analysis. The optimal cut-off values for metabolic parameters were obtained by receiver operating characteristic (ROC) curve.

**Results:**

A total of 20 unicentric CD (UCD) and 73 multicentric CD (MCD) cases were included, with the highest SUV_max_ of Lymph nodes ranged 1.40 ~ 28.18 (median, 4.86). The metabolic parameters (SUV_max_, MLV, TLG, LLR, LMR, SLR) in MCD were significantly higher than those in UCD (*p* < 0.05). There were significant differences in MLV, TLG, LLR and SLR among different histological subtypes (*p* < 0.05). The No. of involved lymph node stations (LNS) and spleen-to-liver ratio (SLR) were significantly correlated with laboratory findings. In univariate and multivariate analyses, SLR (*p* = 0.011; OR value = 14.806) and HGB (*p* = 0.004; OR value = 0.044) exhibited an independent correlation with disease severity. The ROC curve revealed that SLR had a sensitivity of 77.4%, specificity of 69.4% and AUC of 0.761 (cut-off value = 1.04; *p* < 0.001) in discriminating severity of CD. SLR also showed significant statistical differences between severe and non-severe idiopathic MCD (iMCD) (*p* = 0.016).

**Conclusions:**

SLR is closely related to clinical features of CD, and can relatively effectively differentiate the severity of CD and assist in the clinical risk stratification of iMCD.

**Supplementary Information:**

The online version contains supplementary material available at 10.1186/s40644-025-00833-9.

## Background

Castleman disease (CD) is a rare lymphoproliferative disorder that was first described in 1954 [1]. Histologically, CD is classified into three types: the hyaline vascular type (HV), the plasma cell type (PC), and the mixed cell type (Mixed) [2]. Clinically, CD can be classified as unicentric(UCD) or multicentric (MCD) depending on the distribution area of involved lymph nodes. UCD usually presents with slow-growing solitary mass and most patients have no concomitant symptoms. MCD involves enlarged lymph nodes in multiple areas and patients often also present with systemic manifestations such as fever, night sweats, malaise, weight loss, anemia, hepatic insufficiency, renal insufficiency, volume overload (general edema, hydrothorax, ascites, etc.) or multiple organ dysfunction which may be fatal if not treated properly [3, 4]. MCD has a more aggressive clinical course than UCD and is prone to malignant transformation [5–7]. According to the recently published china castleman disease network (CCDN) consensus [8], MCD can be divided into human herpesvirus 8 (HHV-8) associated MCD and HHV-8 negative MCD. HHV-8 negative MCD can be further divided into asymptomatic MCD (aMCD) and idiopathic MCD (iMCD). The former has no systemic symptoms or hyperinflammatory manifestations besides lymph node enlargement, while the latter is accompanied by systemic symptoms and/or organ damage. Excessive pro-inflammatory hypercytokinemia, often including interleukin (IL)-6, is believed to play a role in the pathogenesis of MCD [2, 9, 10].

Before CD treatment, a comprehensive examination is of great significance for risk stratification and prognosis [11–14]. The main assessment methods include: symptom evaluation, imaging examinations, pathogen or immune-related tests, inflammatory status, and organ damage assessment, etc. Some studies have reported the advantages of whole-body PET/CT scans in evaluating the extent of CD involvement [11, 15], and some studies have reported the correlation of ^18^F-FDG PET/CT with clinical features, however, most of the studies were limited to a small number of metabolic parameters such as SUV_max_. Research has confirmed that the inflammatory status and disease severity of CD patients are of significant importance for the staging, diagnosis, and prognosis evaluation of CD [8, 16–18]. However, the value of FDG PET/CT in this context remains unclear.

Our research aims to systematically evaluate the correlation between FDG PET/CT multi- metabolic parameters and clinical typing, pathological typing, laboratory tests, and clinical symptoms. We also aim to explore the value of FDG PET/CT in discriminating severity of CD and assisting in grading of iMCD risk.

## Materials and methods

### Patients

**Fig. 1:**
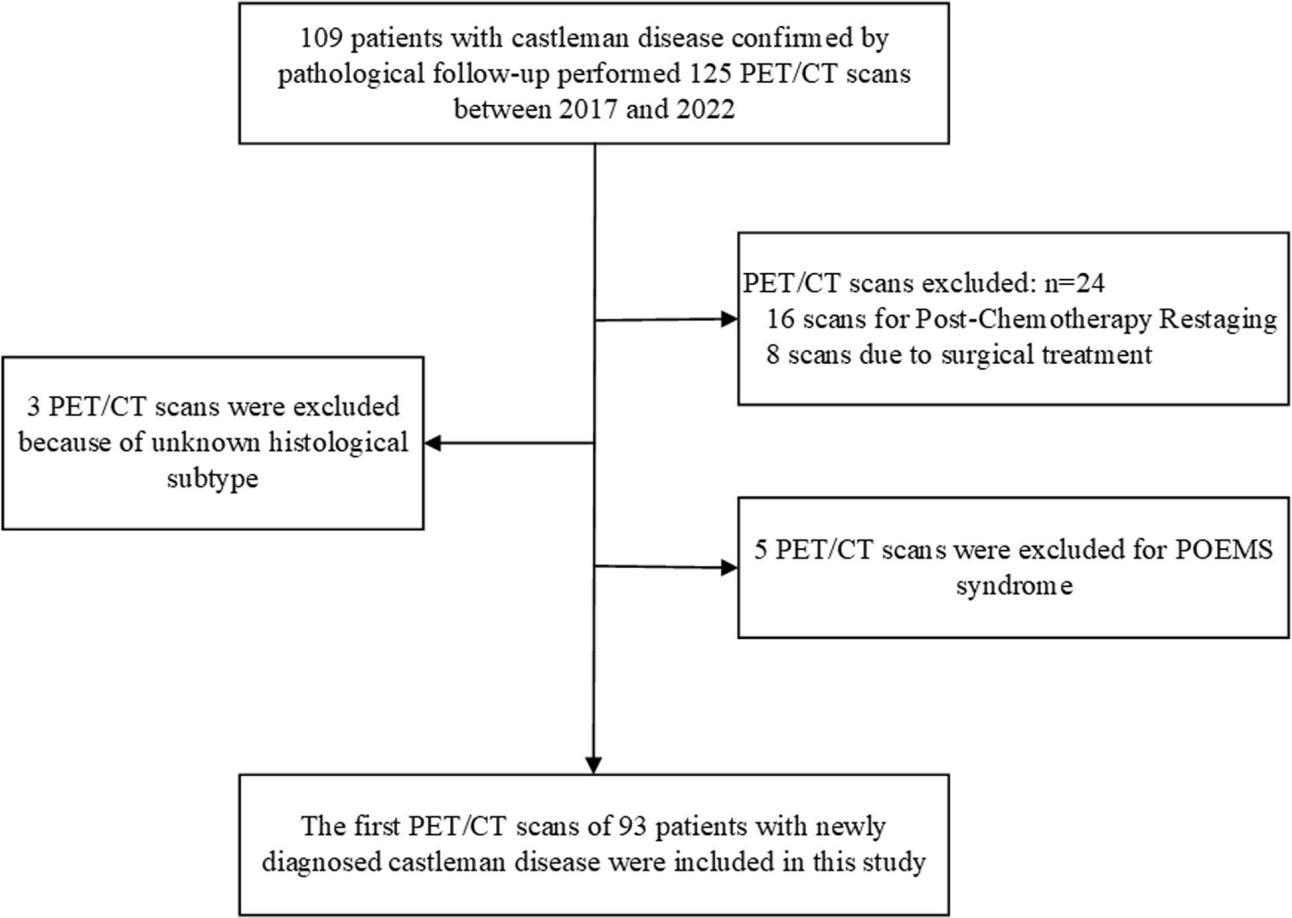
Flowchart of cases inclusion and exclusion

This study enrolled patients newly diagnosed with CD by pathology in our hospital from 2017 to 2023. All patients underwent PET/CT scans before treatment. We retrospectively analyzed the FDG PET/CT images, clinical and pathological data and laboratory findings within one month of the PET/CT scan in patients. The exclusion criteria for this study were: 1. missing important clinical data; 2. having undergone surgery or chemotherapy before PET/CT scan; 3. patients with a confirmed diagnosis of POEMS (Polyneuropathy, Organomegaly, Endocrinopathy, Monoclonal plasma proliferative disorder and Skin changes) CD. The study workflow is displayed in Fig. 1.

### ^18^F-FDG PET/CT imaging

The PET/CT scanners used in this study included Biograph 16 and Biograph Vision 600 (Siemens Healthineers). All patients fasted for at least 6 h and avoided strenuous exercise before ^18^F-FDG injection. The blood glucose was measured and recorded, which should not exceed 11.0 mmol/L. Intravenous injection of ^18^F-FDG (5.55 MBq/kg) was administered, and scanning began approximately 50 ~ 70 min later, and the start time was recorded. All patients were scanned head and body separately, with low-dose CT scans performed first for attenuation correction and anatomical location, followed by PET acquisition at a time of 2 min/bed. The body scan range was from the skull base to the middle of the thigh, about six or seven beds. All PET images were reconstructed using standard iterative reconstruction algorithms.

### Image analysis

The PET/CT images were reviewed retrospectively by two experienced nuclear medicine physicians and the range of CD involvement was recorded, excluding obvious reactive hyperplastic lymph nodes. Each PET/CT scan was divided into 13 lymph node stations according to the involved sites, including bilateral cervical (including supraclavicular fossa), bilateral axillary, mediastinal, bilateral hilar, abdominal, para-aortic, bilateral iliac, and bilateral inguinal. The inclusion criteria for lymph nodes into analysis were CT with a short diameter > 1 cm or < 1 cm but with significantly higher FDG uptake than liver SUV_mean_ [15].

First, we evaluated whether splenomegaly (head-to-tail size > 13 cm), lung lesions or abdominal and pelvic meso-omental thickening were present on CT images. Second, on PET images, right liver SUV_max_ and SUV_mean_ were measured in a 3 cm diameter spherical region of interest (ROI); The SUV_max_ of the mediastinal blood pool was measured the on thoracic aorta, avoiding the vessel wall. We also measured the SUV_max_ of spleen and bone marrow; The lesion ROIs were delineated using a 41% SUV_max_ threshold through combination of semi-automatic and manual [19]; The parameters including SUV_max_, MLV and TLG. MLV was defined as the sum of the volumes of all hypermetabolic lesions. TLG was calculated as the volume of MLV multiplied the SUV_mean_ of MLV. To further explore the correlation between metabolic associations among lymph nodes, liver, spleen, mediastinal blood pool and clinical features, we also calculated parameters such as LLR, LMR, SLR, LNS, respectively. LLR was defined as the lymph node to liver ratio of SUV_max_, LMR was defined as lymph node to mediastinal blood pool of SUV_max_, SLR was defined as the spleen to liver ratio of SUV_max_, and LNS was defined as No. of involved lymph node stations.

### Clinical assessment

According to the NCI common toxicity criteria for adverse events (CTCAE), version 5.0 [20], CD patients were divided into low severity group (Mild; CTCAE grade ≤ 2) and high severity group (Severe; CTCAE grade ≥ 3) [21]. This work evaluated the utility of PET/CT metabolic parameters in differentiating disease severity using univariate and multivariate analysis methods. On the other hand, according to the different clinical symptoms of CD patients, we analyzed the metabolic differences between symptomatic and asymptomatic groups. In addition, among the patients diagnosed with iMCD, we also classified them into severe and non-severe groups based on the risk stratification criteria for iMCD from the castleman disease collaborative network (CDCN) to analyze their metabolic differences [11].

### Statistical analysis

Categorical variables are expressed as counts and percentages of cases, continuous variables that conformed to a normal distribution are expressed as mean ± standard deviation (SD), and continuous variables that did not conform to a normal distribution are expressed as medians with interquartile ranges (IQR). The clinical features of patients between different groups were compared by using T tests, Mann–Whitney U test, Chi-square test and Fisher exact tests as appropriate. The McNemar test was used to compare the differences in the distribution of lymph node involvement areas detected by PET and CT. Binary logistic regression models were employed for univariate and multivariable analysis of disease severity influencing factors. According to the normal value range of the institution, the cut-off values for hemoglobin (HGB) was 120 g/l for men and 110 g/l for women, the cut-off values for platelet (PLT), C-reactive protein (CRP), erythrocyte sedimentation rate (ESR), interleukin 6 (IL-6) and albumin (ALB) were 100,000/ml, 8 mg/l, 15 mm/h, 2.9 pg/ml and 35 g/l. All statistical analyses were performed using SPSS 26.0 and GraphPad Prism 9.0. A two-side *p*-value less than 0.05 was considered statistically significant.

## Results

### Baseline patient characteristics

A total of 93 newly-diagnosed CD patients (49 males; 44 females) were enrolled in the study, with a median age of 53 years (range, 14 ~ 78 years). The average waiting time for PET imaging was 61.1 ± 3.5 min (range, 54 ~ 69 min). The baseline characteristics of patient population are shown in Table 1. The proportions of HV, PC and Mixed are significantly different between UCD (85%, 10% and 5%) and MCD (19%, 63% and 11%), *p* < 0.001. The proportion of patients with clinical symptoms in MCD was significantly higher than that in UCD (*p* < 0.001), and the proportion of IL-6 positives in MCD was significantly higher than that in UCD (*p* = 0.049). Among 48 patients who underwent HHV-8 testing, only 4 (8.3%) were positive for HHV-8, and all were from MCD. In addition, only one of the patients had a history of human immunodeficiency virus (HIV) infection.

**Table 1:** Baseline patient characteristics

**Variable**	**UCD** (n = 20)	**MCD** (n = 73)	***p***
Age (years, median)	46 (23 ~ 76)	54 (14 ~ 78)	0.133
Blood glucose (mmol/L)			0.035
mean ± SD	5.4 ± 1.0	5.9 ± 1.1	
Range	4.0 ~ 9.1	4.0 ~ 8.9	
Gender			0.074
Male	7 (35.0%)	42 (57.5%)	
Female	13 (65.0%)	31 (42.5%)	
Histology type			0.001
Hyaline vascular	17 (85.0%)	19 (26.0%)	
Plasma cell	2 (10.0%)	46 (63.0%)	
Mixed cell	1 (5.0%)	8 (11.0%)	
IL-6			0.049
Positive	7 (70.0%)	59 (90.4%)	
Negative	3 (30.0%)	4 (9.6%)	
Unknown	10	10	
Complications			
Involvement	7 (35.0%)	52 (71.2%)	0.003
No involvement	13 (65.0%)	21 (28.8%)	
Clinical symptoms			
Symptomatic	8 (40.0%)	59 (80.8%)	0.001
B-symptoms	2 (10.0%)	28 (38.4%)	0.012
Splenomegaly	3 (15.0%)	30 (41.1%)	0.036
Oedema/effusion	2 (10.0%)	36 (49.3%)	0.002
Active pneumonia	4 (20.0%)	17 (23.3%)	1.000

*IL-*6 interleukin 6, *B-symptoms* night sweats: fever, drenching, and a weight loss of more than 10% over 6 months; *p* < 0.05: signifcant; *SD* standard deviation

### ^**18**^**F-FDG PET/CT findings **

**Fig. 2:**
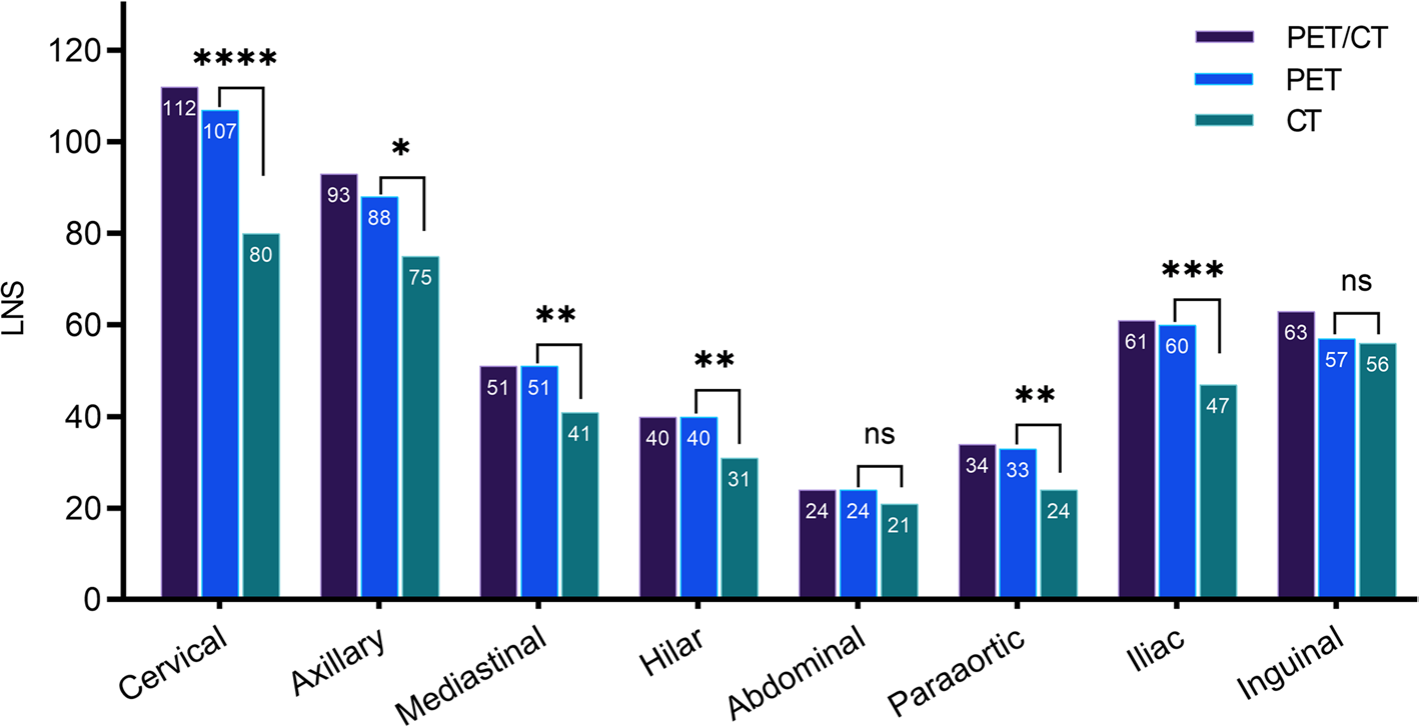
Distribution of involved lymph node stations in castleman disease. Bilateral cervical, axillary, hilar, iliac and inguinal involvement counted twice. LNS: No. of involved lymph node stations

A total of 477 involved lymph node stations were detected by PET/CT, including 375 (78.6%) by CT and 458 (96.0%) by PET. Among the 13 lymph node distribution areas, PET detection capability was significantly better than CT in all areas except for the abdomen and bilateral inguinal regions (Fig. 2). The most common lymph node involvement area in UCD was the cervical (*n* = 9/20; 45%), followed by the mediastinal/hilar (*n* = 3; 15%), axilla (*n* = 3; 15%), abdominal/retroperitoneal (*n* = 4; 20%) and pelvis (*n* = 1; 5%). The most common lymph node stations in MCD were the cervical (*n* = 61/73; 83.6%), axilla (*n* = 51; 69.9%), mediastinal (*n* = 48; 65.8%), inguinal (*n* = 36; 49.3%), iliac (*n* = 35; 47.9%), and paraaortic (*n* = 32; 43.8%), while uncommon lymph node stations were located in the hilar region (*n* = 24; 32.9%) and abdomen (*n* = 22; 30.1%). The median LNS in MCD was 6 (IQR, 7; rang, 2 ~ 13).

For all patients, the maximum short diameter of involved lymph nodes ranged 0.80 ~ 10.90 cm (median, 1.70; IQR, 0.92), and the highest SUV_max_ ranged 1.40 ~ 28.18 (median, 4.86; mean, 5.95 ± 3.80). The proportion of lymph nodes with increased metabolism was 96%. UCD had a significant statistical difference from MCD in FDG metabolic parameters (SUV_max_, *p* = 0.004; MLV, *p* < 0.001; TLG, *p* < 0.001; LLR, *p* < 0.001; LMR, *p* = 0.001; SLR, *p* < 0.001) (Table 2). The metabolic parameters (MLV, *p* = 0.002; TLG, *p* = 0.002; LLR, *p* = 0.024; SLR, *p* < 0.001) were significantly different among histological subtypes (HV, PC and Mixed). In adition, the metabolism of PC was higher than Mixed, and Mixed was higher than HV (Table 3).

**Table 2:** Comparison of metabolic parameters between UCD and MCD

**Variable**	**UCD** (*n* = 20)	**MCD** (*n* = 73)	**Total** (*n* = 93)	***p***
SUV_max_	3.9 (2.2)	5.3 (4.3)	4.9 (4.0)	0.004
MLV	9 (26)	29 (48)	24 (41)	< 0.001
TLG	23 (68)	74 (135)	60 (129)	< 0.001
LLR	1.26 (0.77)	2.00 (1.13)	1.86 (1.06)	< 0.001
LMR	2.26 (1.36)	3.15 (2.08)	2.93 (1.99)	0.001
SLR	0.87 ± 0.15	1.12 ± 0.30	1.07 ± 0.29	< 0.001

*UCD* unicentric castleman disease, *MCD* multicentric castleman disease, *SUV*_*max*_ maximum standardized uptake value, *MLV* metabolic lesion volume, *TLG* total lesion glycolysis, *LLR* lymph node to liver ratio of SUV_max_, *LMR* lymph node to mediastinum ratio of SUV_max_, *SLR* spleen to liver ratio of SUV_max_, *p* < 0.05: signifcant.

**Table 3:** Comparison of metabolic parameters between histologic subtypes

**Variable**	**HV** (*n* = 36)	**PC** (*n* = 48)	**Mixed** (*n* = 9)	***p***
SUV_max_	4.1 (2.6)	5.3 (4.4)	5.0 (4.1)	0.120
MLV	12 (21)	46(46)	24 (34)	0.002
TLG	31(67)	124 (144)	69(123)	0.002
LLR	1.69 (0.88)	2.13 (1.44)	1.92 (1.10)	0.024
LMR	2.49 (1.61)	3.26 (2.35)	2.65 (2.54)	0.059
SLR	0.90 ± 0.17	1.19 ± 0.29	1.07 ± 0.36	< 0.001

*HV* hyaline vascular type, *PC* plasma cell type, *Mixed* mixed cell type, *SUV*_*max*_ maximum standardized uptake value, *MLV* metabolic lesion volume, *TLG* total lesion glycolysis, *LLR* lymph node to liver ratio of SUV_max_, *LMR* lymph node to mediastinum ratio of SUV_max_, *SLR* spleen to liver ratio of SUV_max_, *p* < 0.05: signifcant.

### Metabolic parameters compared to laboratory findings

A total of 6 laboratory tests (HGB, PLT, CRP, ESR, IL-6, ALB) were included in this study to compare the differences in PET/CT metabolic parameters between the normal and abnormal groups (Suppl Table 1). LLR was significantly higher in abnormal groups of inflammatory indicators CRP and ESR than in normal groups (*p* = 0.031 & *p* = 0.021); TLG was significantly higher in abnormal groups of PLT and ESR than in normal groups (*p* = 0.010 & *p* = 0.004); The LNS was significantly higher in abnormal groups of all laboratory tests than in the normal groups (*p* < 0.05); while SLR was significantly higher in abnormal groups of HGB, PLT, CRP, ESR and ALB than in normal groups (*p* < 0.01).

### Comparison of PET/CT findings and clinical symptoms

**Fig. 3:**
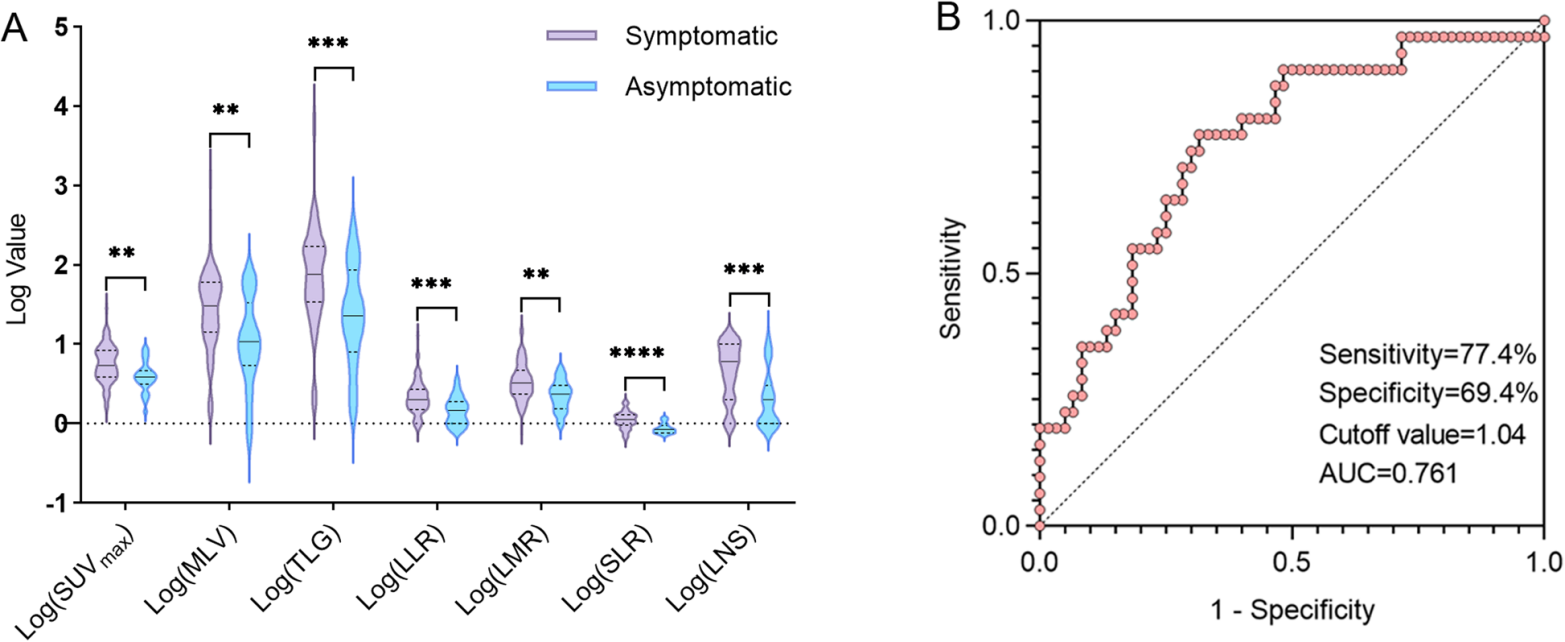
**A** Metabolic differences between symptomatic and asymptomatic groups statistical chart. The logarithm to the base 10 of SUV_max_, MLV, TLG, LLR, SLR, and LNS was taken respectively. The differences between the symptomatic group and the asymptomatic group were compared. **B** Receiver operating characteristic (ROC) curve analysis for SLR to predict the severity of castleman disease

In 93 patients, 67 (72.0%) had clinical symptoms and 26 (28.0%) were asymptomatic (Table 1). In symptomatic patients, 30 (32.3%) were present with B symptoms, 33 (35.5%) with splenomegaly, 38 (40.9%) with serous cavity effusion and 65 (69.9%) with CT manifestations of lung cysts, nodules, thickening of the bronchovascular bundle or interlobular septal thickening, but only 21 (22.6%) patients had increased FDG uptake, with a SUV_max_ ranged 1.1 ~ 10.0 (median, 2.1). A small number of patients presented with pain, rash or digestive system symptoms. Figure 3A showed that the metabolic parameters of symptomatic group were significantly higher than those of asymptomatic group (SUV_max_, *p* = 0.001; MLV, *p* = 0.002; TLG, *p* < 0.001; LLR, *p* < 0.001; LMR, *p* = 0.002; SLR, *p* < 0.001; LNS, *p* < 0.001; MLV, *p* = 0.002). In addition, 15 (16.1%) patients had combined abdominal pelvic omental, mesenteric thickening or perirenal fascia with fuzzy density, of whom 8 (8.6%) had mild increase of FDG uptake; 9 (9.7%) had bilateral parotid hyper-metabolism; 5 (5.4%) had unilateral or bilateral adrenal hyper-metabolism foci; 3 (3.2%) had FDG uptake foci in liver, one case of whom was proved rich lympho-plasmacytic infiltration by liver puncture biopsy; 2 (2.2%) had FDG uptake foci in pancreas; 2 females had increased FDG uptake in the myometrium or adnexa, one of whom was diagnosed with uterine fibroids without plasma cell infiltration by postoperative pathology; one patient with gastric leiomyoma had increased FDG metabolism at the tumor margin, and was confirmed massive lympho-plasmacytic infiltrations outside the serosal layer after surgery. However, most of the extranodal organs uptake foci failed to obtain pathological data.

### The value of PET/CT in differentiating the severity of CD

**Fig. 4:**
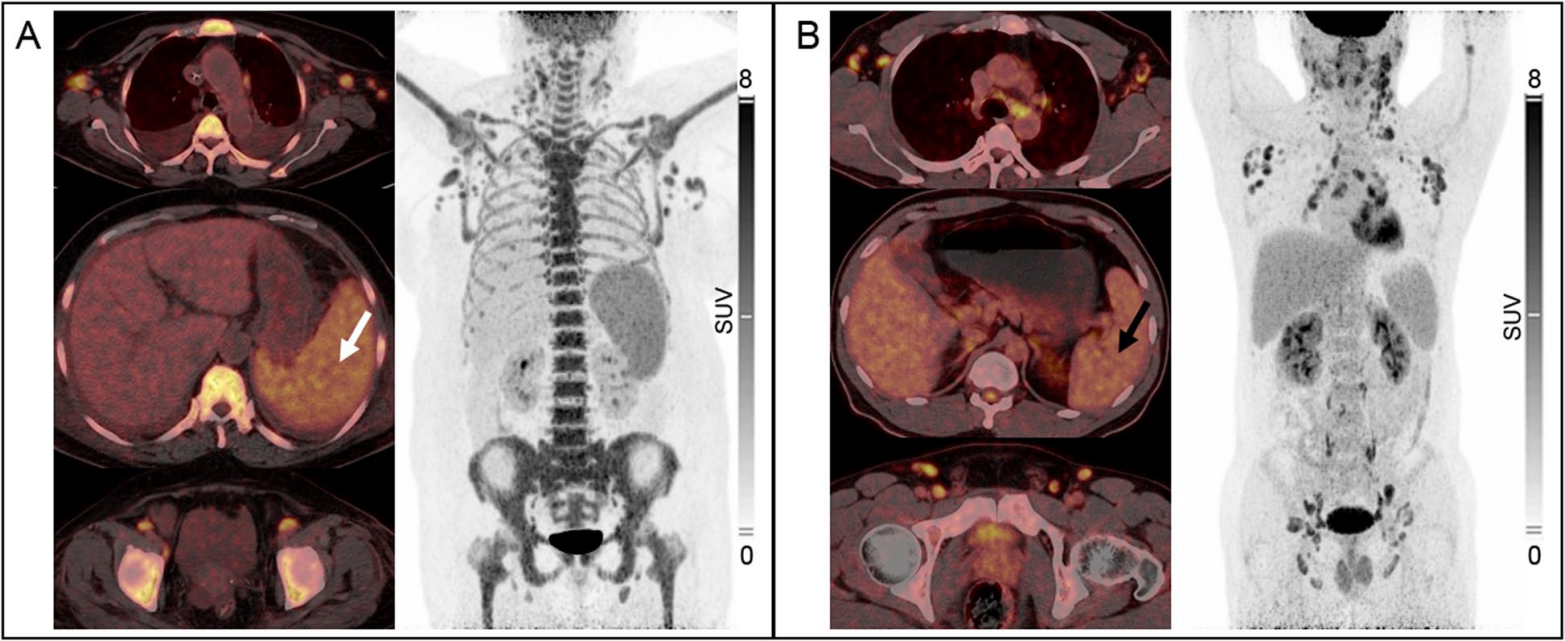
**A** High severity case: A 67-years-old female, admitted to the hospital with asthenia, abdominal pain and diarrhea. She was finally diagnosed as plasma cell type iMCD, disease severity grade 4 (as per CTCAE 5.0 standards), and the iMCD risk stratification was severe (as per CDCN standards). PET/CT showed multiple serous cavity effusion, multiple enlarged lymph nodes in the whole body with increased FDG uptake, diffused increased metabolism in the bone marrow and increased metabolism in the spleen (SUV_max_ 5.03, as indicated by the white arrow), and the SLR was 1.99 (SLR > 1.04). **B** Low severity case: A 33-year-old male was admitted to the hospital due to abnormal renal function and lower limb edema. He was ultimately diagnosed with hyaline-vascular type iMCD, with a CTCAE grade of 0, and the risk stratification for iMCD was non-severe. PET/CT revealed multiple enlarged lymph nodes throughout the body accompanied by increased FDG uptake. the spleen showed no high metabolism (SUV_max_ 3.3, as indicated by the black arrow), and the SLR was 0.83 (SLR < 1.04)

To explore the influencing factors of disease severity in CD patients, this study included 7 metabolic parameters and 7 clinical risk factors. The univariate analysis revealed that there were significant statistical differences between the severe group (*n* = 31) and the mild group (*n* = 62) in terms of SLR (*p* < 0.0001), LNS (*p* < 0.001), hypohemoglobinemia (*p* < 0.0001), hypoalbuminemia (*p* < 0.0001), splenomegaly (*p* = 0.002), serous cavity ascites (*p* = 0.002) and age (*p* = 0.018) (Table 4). In the binary logistic regression analysis, stepwise regression method (forward LR) was used to screen variables with statistical differences, and finally SLR (*p* = 0.011, OR value = 14.8) and hypohemoglobinemia (*p* = 0.004, OR value = 0.044) were included as independent influencing factors with disease severity (Table 5). The subsequent receiver operating characteristic (ROC) curve revealed that SLR can relatively effectively judge the severity of CD, with a sensitivity of 77.4%, specificity of 69.4%, cut-off value of 1.04 and area under the curve (AUC) of 0.761 (Fig. 3B). In addition, to further study the relationship between SLR and high-risk iMCD, according to the stratified criteria of iMCD risk level by CDCN, a total of 33 patients (19 severe and 14 non-severe) with iMCD were included in this study, and the independent sample T test showed that the SLR value of patients with severe iMCD was significantly higher than that of non-severe (*P* = 0.016) (Fig. 4A, 4B).

**Table 4:** Comparison of risk factors between mild group and severe group

**Factor**	**Mild** (*n* = 62)	**Severe** (*n* = 31)	***p***
SUV_max_	4.7 (3.7)	5.1 (4.6)	0.903
MLV	20(43)	39 (47)	0.172
TLG	54 (111)	125(158)	0.244
LLR	1.84 (1.06)	2.01 (1.40)	0.728
LMR	2.91 (1.69)	3.21 (2.60)	0.669
SLR	0.98 ± 0.22	1.25 ± 0.33	< 0.001
LNS	3 (6)	7 (6)	< 0.001
Age (year)	48 (24)	56 (25)	0.018
Sex (m/f)	32/28	16/15	0.877
Spleen size (cm)	11.0 ± 2.8	12.9 ± 2.8	0.002
HGB decrease	23(37.1%)	30(96.8%)	< 0.001
ALB decrease	19(30.6%)	27(87.1%)	< 0.001
serous effusion	18(29.0%)	20(64.5%)	0.002
Pneumonitis	49(64.5%)	25(80.6%)	0.110

*SUV*_*max*_ maximum standardized uptake value, *MLV* metabolic lesion volume, *TLG* total lesion glycolysis, *LLR* lymph node to liver ratio of SUV_max_, *LMR* lymph node to mediastinum blood pool ratio of SUV_max_, *SLR* spleen to liver ratio of SUV_max_, *LNS* No. of involved lymph node stations, *HGB* hemoglobin, *ALB* albumin, *y* years, *cm* centimeter, *m* male, *f* female, *p* < 0.05: signifcant.

**Table 5:** Binary logistic regression analysis of influencing factors of CD severity

Factor	*p*	OR value	95% CI
SLR	0.011	14.806	1.833 ~ 119.597
HGB decrease	0.004	0.044	0.005 ~ 0.366

*SLR* spleen to liver ratio of SUV_max_, *HGB* hemoglobin, *OR* odds ratio, *CI* confidence interval, *p* < 0.05: signifcant.

## Discussion

To date, our study included the largest sample of FDG PET/CT scans in newly-diagnosed CD patients. Some previous studies have explored the relationship between FDG PET/CT and clinical symptoms, clinico-histotyping or laboratory examinations in CD patients [22, 23], however, these studies mainly focused on a single metabolic parameter, and most of them had small sample sizes. To fully explore the clinical application value of FDG PET/CT in CD, this study expanded the range of metabolic parameters. To fully explore the clinical application value of FDG PET/CT in CD, this study expanded the range of metabolic parameters and analyzed the correlation between FDG metabolic parameters and clinical characteristics as well as disease severity. It was found that the SLR had better performance than other metabolic parameters and was independently correlated with disease severity.

Recently, a multicenter retrospective study of 1634 CD patients in China [16] showed that UCD was more common than MCD; In UCDs, the HV accounted for 79.0%, while the PC accounted for 52.3% in MCDs. In our study, the proportion of UCD patients was lower than that of MCD, possibly because MCD is more likely to present with systemic symptoms and persistent diseases, making it more likely to undergo PET/CT examination [22–24]; on the other hand, the proportions of histological subtypes of UCD and MCD in this study were consistent with the above results.

In this study, the distribution of involved lymph node area was consistent with the reported in the literature [22], and PET was better than CT for the detection capability of involved lymph nodes (96% vs 78.6%), which was consistent with the result of Barker et al. [15]. Whole-body PET/CT imaging is also of great significance for the evaluation of complications, and this study showed that 35.0% of UCD and 71.2% of MCD patients presented with complications such as splenomegaly, serous cavity effusion, active lung inflammation and thickening of the omentum mesentery. Jiang et al. [25] reported that 60% of MCD cases were found to have multiple cystic changes in the lungs. Our study showed similar results but only 21 (22.6%) patients had manifestations of active inflammation.

The patient population in this study exhibited moderate FDG uptake with a mean highest SUV_max_ of 5.95 (range, 1.40 ~ 28.18; median, 4.86), which was generally consistent with several other studies [23–25]. Previous studies have shown that there was a significant statistical difference in SUV_max_ between UCD and MCD groups, as well as symptomatic and asymptomatic groups, but no statistical difference among histological subtypes [22, 23]. According to our study, all metabolic parameters (SUV_max_, MLV, TLG, LLR, LMR and SLR) were significantly statistically different between UCD and MCD groups, as well as symptomatic and asymptomatic groups, which was similar to previous studies; On the other hand, unlike previous studies, MLV, TLG, LLR and SLR showed significant statistical differences among histological subtypes, and PC-CD had higher metabolism than HV-CD, which indicated that PC-subtype was more invasive to some extent.

For UCD, the preferred surgical complete resection of lesions is used regardless of whether the patient has a hyperinflammatory state or systemic symptoms [1, 26–28]; for HHV-8 positive MCD, rituximab-based therapy can be adopted [29–31]; for asymptomatic and non-hyperinflammatory aMCD, observation with follow-up is primary; for symptomatic and hyperinflammatory iMCD, different treatment strategies are adopted according to the non-severe and severe categories defined by the CDCN risk stratification [2, 3, 11]. The inflammatory status plays a significant role in both CD stratification and efficacy evaluation. The core therapeutic goal of iMCD is to control the hyperinflammatory state, not lymph node size [8, 17, 18]. According to our study, CD patients with a high inflammatory state had significantly higher LLR, LNS and SLR, which illustrated that patients with a high inflammatory state exhibited higher metabolic activity of lymph nodes and spleens, as well as more involved lymph node stations. In addition, It has been reported that PC-subtype, hepatosplenomegaly, Hb ≤ 80 g/L and Alb ≤ 30 g/L were independently associated with CD overall survival (OS) [32, 33]. In our study, patients with hypohemoglobinemia and hypoalbuminemia presented with more involved lymph node stations and significantly elevated SLR values. These findings suggest that FDG PET/CT can complement laboratory findings to assess disease activity in CD patients and help with prognosis analysis.

In a study of Kaposi's sarcoma herpesvirus associated MCD (KSHV-MCD) by Polizzotto et al. [21], the severity of KSHV-MCD in active disease was correlated with the intensity of lymph node SUV_max_. However, in our study, SUV_max_ did not show a correlation with disease severity, possibly due to the differences in enrolled cases, because about 92% of the KSHV-MCD cases they reported were HIV positive, while in our study, only one patient had a history of HIV infection and was not a KSHV-MCD patient. On the other hand, we identified SLR and hypohemoglobinemia as independent influencing factors for CD severity through univariate and multivariate analyses. Recently, Zhang et al. [16] study confirmed the rationality of the concept of severe iMCD proposed by CDCN. Our study found that the SLR value of severe iMCD was significantly higher than that of non-severe iMCD. As a rare lymphoid hyperplasia disease, the role of FDG PET/CT in CD prognosis is not yet clear, and our study showed that FDG PET/CT may assist in risk stratification for patients with iMCD.

This study has several limitations. Firstly, this is a single-center study and CD as a rare disease with relatively few cases, so there may be selection bias in this study. Secondly, as this study was retrospective and many MCD patients lacked HHV-8 test results, only a small number of cases were included in our study of severe and non-severe iMCD, which needs further investigation. At last, the study did not collect enough survival data to perform prognostic analysis. In future work, we plan to continue follow-up with patients and improve the value of FDG PET/CT in CD prognosis.

## Conclusion

In summary, the metabolic parameters of ^18^F-FDG PET/CT are significantly correlated with the clinical and pathological classification, laboratory tests and clinical symptoms of CD patients. SLR can relatively effectively differentiate the severity of CD and assist clinicians in risk stratification of severe and non-severe categories in iMCD patients.

## Supplementary Information

Additional file 1.

## Data Availability

No datasets were generated or analysed during the current study.

## Abbreviations

ALB: Albumin
CD: Castleman disease
CDCN: Castleman disease collaborative network
CRP: C-reactive protein
CTCAE: Common toxicity criteria for adverse events
ESR: Erythrocyte sedimentation rate
FDG: Fluorodeoxyglucose
HGB: Hemoglobin
HHV-8: Human herpesvirus 8
HIV: Human immunodeficiency virus
HV: Hyaline vascular type
IL-6: Interleukin 6
iMCD: Idiopathic MCD
IQR: Interquartile ranges
LLR: Lymph node to liver ratio of SUV_max_
LMR: Lymph node to mediastinal blood pool of SUV_max_
LNS: No. of involved lymph node stations
MCD: Multicentric castleman disease
Mixed: Mixed cell type
MLV: Total lesion metabolic volume
PC: Plasma cell type
PET/CT: Positron emission tomography/computed tomography
PLT: Platelet
ROC: Receiver operating characteristic curve
SLR: Spleen to liver ratio of SUV_max_
SUV_max_: Maximum standardized uptake value
TLG: Total lesion glycolysis
UCD: Unicentric castleman disease
