# Supplementary material for: 18F-FDG PET/CT metabolic parameters are correlated with clinical features and valuable in clinical stratification management in patients of castleman disease
Source: Cancer Imaging. 2025 Feb 12;25:12. doi: 10.1186/s40644-025-00833-9 (PMC11823125; doi:10.1186/s40644-025-00833-9)
Supplement: Supplementary file 1 — Additional file 1. [file 40644_2025_833_MOESM1_ESM.docx]

| **Suppl Table 1** Comparison of metabolic parameters between normal and abnormal laboratory findings | | | | | | | | | |
| --- | --- | --- | --- | --- | --- | --- | --- | --- | --- |
|  | | **LLR**  median (IQR) | **P-value** | **TLG**  median (IQR) | **P-value** | **LNS**  median (IQR) | **P-value** | **SLR**  mean±SD | **P-value** |
| HGB | Low  Normal | 1.96 (1.06)  1.71 (1.24) | 0.536 | 75.0 (138.3)  38.4 (105.4) | 0.153 | 7 (7)  2 (2) | <0.001 | 1.16±0.28  0.92±0.23 | <0.001 |
| PLT | Low  Normal | 1.92 (1.08)  1.84 (1.28) | 0.606 | 121.9 (148.9)  43.2 (111.8) | 0.011 | 7 (7)  2 (5) | <0.001 | 1.16±0.32  1.01±0.25 | 0.001 |
| CRP | Elevated  Normal | 2.16 (1.38)  1.71 (1.14) | 0.031 | 105.0 (131.7)  51.1 (116.4) | 0.088 | 7 (8)  3 (6) | 0.002 | 1.19±0.31  0.95±0.19 | <0.001 |
| ESR | Elevated  Normal | 2.13 (1.53)  1.66 (1.08) | 0.021 | 125.8 (144.7)  41.1 (73.5) | 0.004 | 7 (7)  2 (4) | <0.001 | 1.22±0.29  0.96±0.26 | <0.001 |
| IL-6 | Elevated  Normal | 2.00 (1.09)  1.89 (1.36) | 0.431 | 80.3 (151.1)  42.5 (25.4) | 0.149 | 6 (7)  2 (5) | 0.031 | 1.10±0.29  0.97±0.27 | 0.252 |
| ALB | Low  Normal | 1.94 (1.09)  1.86 (1.28) | 0.637 | 96.6 (136.9)  42.5 (106.7) | 0.193 | 7 (6)  2 (4) | <0.001 | 1.20±0.30  0.94±0.22 | <0.001 |
| HGB: hemoglobin; PLT: platelet; CRP: C-reactive protein; ESR: erythrocyte sedimentation rate; IL-6: interleukin 6; ALB: albumin; LLR: lymph node to liver ratio of SUV_max_; TLG: total lesion glycolysis; LNS: No. of involved lymph node stations; SLR: spleen to liver ratio of SUV_max_; P<0.05: signifcant | | | | | | | | | |

**^18^F-FDG PET/CT metabolic parameters are correlated with clinical features and valuable in clinical stratification management in patients of castleman disease**
